# Medial temporal lobe contributions to resting-state networks

**DOI:** 10.1007/s00429-021-02442-1

**Published:** 2022-01-18

**Authors:** Sara Seoane, Cristián Modroño, José Luis González-Mora, Niels Janssen

**Affiliations:** 1 Department of Cognitive, Social and Organizational Psychology, Faculty of Psychology and Speech Therapy, University of La Laguna, San Cristóbal de La Laguna, Spain; 2 Department of Basic Medical Sciences, Faculty of Health Sciences, University of La Laguna, San Cristóbal de La Laguna, Spain; 3 Institute of Biomedical Technologies, University of La Laguna, San Cristóbal de La Laguna, Spain; 4 Instituto Universitario de Neurociencia, University of La Laguna, San Cristóbal de La Laguna, Spain

**Keywords:** Medial temporal lobe, Functional connectivity, Resting-state fMRI, Independent component analysis, Dual regression

## Abstract

The medial temporal lobe (MTL) is a set of interconnected brain regions that have been shown to play a central role in behavior as well as in neurological disease. Recent studies using resting-state functional magnetic resonance imaging (rsfMRI) have attempted to understand the MTL in terms of its functional connectivity with the rest of the brain. However, the exact characterization of the whole-brain networks that co-activate with the MTL as well as how the various sub-regions of the MTL are associated with these networks remains poorly understood. Here, we attempted to advance these issues by exploiting the high spatial resolution 7T rsfMRI dataset from the Human Connectome Project with a data-driven analysis approach that relied on independent component analysis (ICA) restricted to the MTL. We found that four different well-known resting-state networks co-activated with a unique configuration of MTL subcomponents. Specifically, we found that different sections of the parahippocampal cortex were involved in the default mode, visual and dorsal attention networks; sections of the hippocampus in the somatomotor and default mode networks; and the lateral entorhinal cortex in the dorsal attention network. We replicated this set of results in a validation sample. These results provide new insight into how the MTL and its subcomponents contribute to known resting-state networks. The participation of the MTL in an expanded range of resting-state networks is in line with recent proposals on MTL function.

**Supplementary Information:**

The online version contains supplementary material available at 10.1007/s00429-021-02442-1.

## Introduction

The medial temporal lobe (MTL) has received much interest in research and the clinic due to its key implication in memory processes (e.g., Alvarez and Squire 1994; Suzuki and Amaral 2004; Squire et al. 2007), as well as due to its involvement in several relatively common pathological conditions (e.g., temporal lobe epilepsy, schizophrenia and Alzheimer’s disease; Douw et al. 2015; Seidman et al. 2003; Kenkhuis et al. 2019; Govindpani et al. 2020). The MTL encompasses a number of different anatomical structures, primarily the parahippocampal and entorhinal cortices as well as the hippocampal formation. Recent resting-state fMRI (rsfMRI) studies have attempted to understand the MTL by considering its functional connectivity with the rest of the brain (Kahn et al. 2008; Libby et al. 2012; Qin et al. 2016; Ranganath and Ritchey 2012; Ritchey et al. 2015; Ruiz-Rizzo et al. 2020; Schröder et al. 2015; Wang et al. 2016). An on-going debate regarding this issue concerns the different whole-brain functional networks that connect to the MTL. Specifically, whereas the traditional view is that the MTL interacts mainly with two whole-brain networks (Kahn et al. 2008; Libby et al. 2012; Qin et al. 2016; Ranganath and Ritchey 2012; Ritchey et al. 2015; Schröder et al. 2015; Barnett et al. 2019), other recent studies using data-driven techniques have found that the MTL connects with additional networks (Ruiz-Rizzo et al. 2020; Wang et al. 2016; Plachti et al. 2019). Characterizing the whole-brain functional networks that co-activate with the MTL has implications for understanding its role in health and disease. One potential reason for why this issue remains unresolved may be due to methodological limitations in previous studies. Here, we relied on a data-driven parcellation of the MTL using the whole-brain high spatial resolution 7T rsfMRI dataset from the Human Connectome Project (HCP).

The standard view on the connectivity between the MTL and the rest of the brain is that the MTL is connected with two distinct whole-brain networks. Both anatomical and functional connectivity studies have shown that MTL connectivity is largely organized along a posterior–anterior gradient. For example, tract-tracing studies in monkeys and rodents have found that posterior sections of the parahippocampal gyrus (PHG) and posterior sections in the hippocampal formation show increased (mono- or poly-synaptic) connectivity with posterior midline regions like the retrosplenial cortex and posterior cingulate cortex, whereas anterior sections of the parahippocampal cortex and anterior sections of the hippocampal formation show increased connectivity with anterior brain regions like the orbitofrontal cortex and amygdala (Aggleton 2012; Jones and Witter 2007; Kobayashi and Amaral 2007, 2003; Kondo et al. 2005; Rosene & Van Hoesen 1977; Strange et al. 2014; Suzuki and Amaral 1994). Similarly, lateral sections of the entorhinal cortex (Ent) have been associated with the posterior whole-brain network, whereas medial Ent has been associated with the anterior network (Jones and Witter 2007; Strange et al. 2014). More recent functional connectivity studies using rsfMRI have further confirmed this bipartite organization of MTL whole-brain connectivity. Specifically, Libby et al. (2012) found that seeds placed in posterior PHG revealed co-activity with posterior midline regions like the retrosplenial cortex, precuneus, posterior cingulate and occipital cortex, whereas seeds placed in more anterior locations produced co-activity with orbitofrontal cortex and inferior temporal cortex. Other studies placing seeds in various locations along the hippocampal long axis have revealed a similar separation of co-activity between posterior and anterior networks (Kahn et al. 2008; Qin et al. 2016), as have studies examining the entorhinal cortex (Schröder et al. 2015). In short, evidence from various sources now confirms that MTL connectivity can be associated with both a posterior and an anterior network (Ranganath and Ritchey 2012; Ritchey et al. 2015).

However, three recent functional connectivity studies have presented results that have challenged this view. These studies have relied on data-driven approaches to examine functional connectivity thereby avoiding potential biases inherent in functional connectivity techniques that rely on placing seeds (Zuo et al. 2010). Specifically, Wang et al. (2016) examined the slice-by-slice connectivity between both the parahippocampal gyrus as well as the hippocampus and the rest of the brain using a hierarchical clustering technique. Interestingly, they found that there were three connectivity clusters along the parahippocampal long axis, one in posterior PHG that connected to the aforementioned posterior network, one in anterior PHG (termed perirhinal cortex) that connected to the anterior network, and one in an even more anterior PHG location that connected to a network of regions that included the insula, post central gyrus and amygdala. Similarly, Ruiz-Rizzo et al. (2020) examined functional connectivity of the MTL as well as the amygdala using a spatially restricted Independent Component Analysis (ICA) approach (Blessing et al. 2016; Formisano et al. 2004). They found that clusters of activity detected inside the MTL and amygdala co-activated with sets of brain regions that correlated with the reference networks of Allen et al. (2011). Specifically, in addition to the default mode network (correlation varied from $r=0.12$ to $r=0.48$ for different MTL activity clusters), they also found that MTL was somewhat connected to other networks like the salience ($r=0.14$), frontal ($r=0.11$), basal ganglia ($r=0.40$) and visual networks ($r=0.11$). Finally, a recent paper by Plachti et al. (2019) focused on the hippocampus using a consensus clustering technique and found that connectivity between the hippocampus and the rest of the brain was best described by 3, 5 and even 7 clusters.

Thus, it appears that whereas some studies have found that the MTL is connected to two different whole-brain functional networks, others have found it is connected to additional different networks. This empirical discrepancy may be the result of methodological limitations in the aforementioned studies. First, previous studies have relied on relatively low spatial resolution fMRI acquisition protocols ($\sim 3.5$ mm voxels). One concern with such low resolution data is that the large size voxels may be unable to accurately separate signals from different resting-state networks leading to variability in the reported results. Second, previous studies have not sufficiently taken into account the observation that the MTL is affected by local magnetic field inhomogeneities that lead to reduced temporal signal-to-noise ratios (tSNR; e.g., Olman et al. 2009; Weiskopf et al. 2006). The additional noise in the MTL region may hamper the detection of MTL contributions to resting-state networks and also produce inconsistencies in the reported results. In this study, we addressed these two limitations in three ways. First, we addressed the separability of signals inside the MTL using a dataset with high spatial resolution (1.6 mm isotropic) acquired at a high field strength (7T). Previous studies have shown that compared to lower resolutions at 3T, increased spatial resolution at 7T results in more clearly defined resting-state networks (Vu et al. 2017). Second, previous studies have also shown that reducing the voxel size in fMRI data avoids partial voluming effects and leads to improved signal detection in areas with low tSNR (Hyde et al. 2001; Robinson et al. 2004; Sladky et al. 2013). Finally, we relied on the spatially restricted group ICA technique (srICA; Blessing et al. 2016; Ezama et al. 2021; Formisano et al. 2004). Whole-brain ICA is frequently used to separate signal from noise in fMRI studies (e.g., Janssen and Mendieta 2020; Smith et al. 2013). Instead, by applying ICA to a particular brain region, the noise profile that is specific to that brain region will be taken into account and result in a more sensitive separation of signal from noise in that region.

The current study relied on high spatial resolution data as well as a targeted analysis approach to clarify the contributions of the MTL in existing resting-state networks. Specifically, whole-brain functional connectivity maps (FC maps) associated with the MTL clusters found by the srICA were computed using the Dual Regression technique (Nickerson et al. 2017). These whole-brain FC maps reflected the large-scale co-activity with the specific MTL clusters found in the previous step. Next, MTL clusters were classified as signal or noise on the basis of an algorithm that relied on the correlation with the 7 known resting-state networks obtained by Yeo et al. (2011). We then used linear mixed effect regression analyses to calculate the relative contribution of each MTL subcomponent in the different resting-state networks. Specifically, the MTL was segmented into anterior and posterior portions of the PHG, head, body and tail of the hippocampus and mEnt and lEnt. Finally, we relied on a test-validation approach in which results obtained in the test dataset were validated on a second dataset.

## Methods

### Participants

Data for all participants were downloaded from the Human Connectome Website. The initial dataset consisted of 184 participants who had participated in the 7T data acquisition. However, data from 12 participants were excluded due to the presence of specific Quality Control issues identified by the HCP (i.e., QC issues A, B, C, and D). The final sample, therefore, consisted of 172 participants, between the ages of 22 and 35 (104 females). Further detailed description on the study subjects may be found in Van Essen et al. (2012). The data analyses were conducted in agreement with the declaration of Helsinki and with the protocol established by the Ethics Commission for Research of the Universidad de La Laguna, the Comité de Ética de la Investigación y Bienestar Animal.

### Data acquisition and preprocessing

Data packages herein used come from the WU-Minn HCP Data-1200 Subjects data set. For this experiment, we downloaded 7T Resting-State fMRI 1.6 mm/32k FIX-Denoised (Compact) and Resting-State fMRI FIX-Denoised (Extended) datasets. As per the HCP reference manual, these data were acquired by the Washington-University and Minnesota Consortium, with a Siemens Magnetom 7T MR Scanner and a Nova32 32-channel Siemens receive head coil from Nova Medical. Four 16-min-long rsfMRI acquisitions were acquired per subject. RsfMRI acquisitions alternated the direction of the phase encoding gradient, where two sessions were acquired in the posterior-to-anterior phase direction and the other two in anterior-to-posterior phase direction. For the resting-state acquisitions, participants were instructed to fix their sight on a white cross-hair over a dark background (Smith et al. 2013). MRI scanning parameters for the resting-state data were based on acquisitions of Gradient-Echo EPI volumes. Each volume contained 85 slices that were acquired with a multiband factor of 5. Slice thickness was 1.6 mm with no gap, the FOV was 208 $\times$ 208 mm, matrix size 130 $\times$ 130, resulting in 1.6 mm isotropic voxels. The TR was 1000 ms, echo time (TE) 22.2 ms, and the flip angle 45$^{\circ }$. We used the first two of these four rsfMRI datasets as a test set (32 minutes of resting-state data), and the last two datasets as a validation set. Both test and validation datasets had alternating phase directions.

The downloaded fMRI datasets consisted of already pre-processed functional data according to HCP minimal preprocessing pipelines ( Glasser et al. 2013). Briefly, transformations that reduce head motion were estimated using FSL MCFLIRT (Jenkinson et al. 2002), fieldmap and gradient distortion corrections were applied, and transformations from fMRI space to MNI space were estimated using non-linear transformations. Importantly, smoothing of the data was minimized in two ways: First, the transform from native to MNI space preserved the native space resolution of the fMRI acquisitions, and second, all transformations (motion correction, fMRI to MNI space) were postponed, combined and applied in a single step using sinc interpolation. Next, the data in MNI space were temporally filtered using a 2000-s high-pass filter and automatically denoised using the FIX program (Griffanti et al. 2014; Salimi-Khorshidi et al. 2014). This program uses semi-automatic classification of head-motion and other artifacts which minimized the potential impact of head-motion artifacts in our data (Salimi-Khorshidi et al. 2014). The final files were demeaned and had native 1.6 mm isotropic resolution in MNI space. We then extracted and regressed out the CSF and WM signal that was obtained from each participant’s wmparc file.

In addition, for the structural data, we downloaded the 3T Structural Preprocessed and 3T Structural Preprocessed Extended packages. Again as per the HCP reference manual, the T1w images were acquired using a 3DMPRAGE protocol TI/TR/TE: 1000/2400/2.14 ms, flip angle = 80$^{\circ }$, resulting in 0.7 mm isotropic voxels. The T2w images were acquired using a 3D T2-SPACE protocol TR/TE: 3200/565 ms, flip angle = variable, and also resulting in 0.7 mm isotropic voxels. The structural images were acquired on a 3T Siemens Connectom Skyra scanner. The downloaded structural packages contained the T1w and T2w images for each participant as well as the full Freesurfer output and transformation matrices that were relevant for our downstream analyses (see below). For additional specific information on the pre-processing of these structural images, we refer to Glasser et al. (2013).

### Data analysis

The aim of this study was to explore the different whole-brain networks that co-activate with the MTL as well as how the different MTL subcomponents contribute to these different networks. To approach these objectives, our analyses were divided into four main steps. A graphical representation of the workflow is displayed in Supplementary Fig. S1. The most relevant results from these analyses will be made available on our github page (https://github.com/iamnielsjanssen).

#### Segmentation of MTL into subcomponents

The first step of our analysis involved the segmentation of the MTL into a number of subcomponents. This segmentation took place in a participant-specific manner, meaning that each segmentation took into account the unique shape of the MTL in each participant’s brain. The MTL subcomponents were the head, body and tail of the hippocampus (hHi, bHi, and tHi), the anterior (aPHG) and posterior (pPHG) parahippocampal gyrus, as well as the medial and lateral entorhinal cortex (lEnt and mEnt). All segmentations relied on the Desikan–Killiany cortical Atlas as well as the subcortical segmentation that is produced by Freesurfer and that was included with the downloaded dataset (i.e., the aparc+aseg atlas in Freesurfer terminology; Desikan et al. 2006). This provides an automatic segmentation of the brain in terms of a set of 42 brain regions that are fitted to the unique morphology of each participant’s brain. This is achieved by combining prior information about the probable spatial location of a given brain area and its surrounding structures with information about the morphology of a specific target participant brain. This way of segmenting the brain into regions is, therefore, more accurate than other atlas segmentations that are based on normalized brains.

**Fig. 1:**
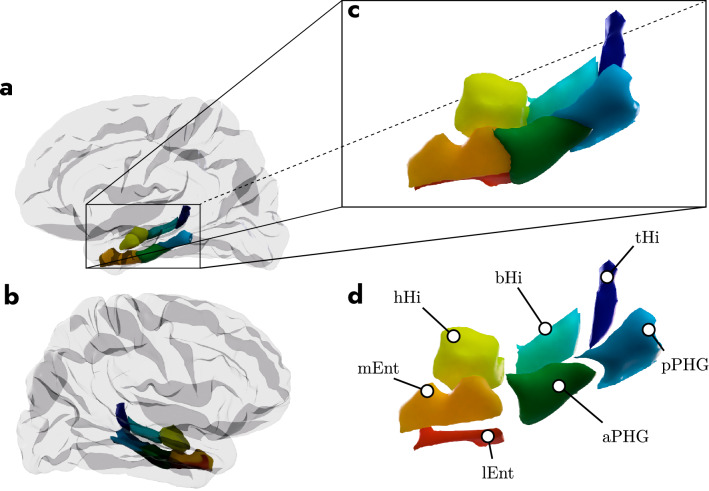
Visualization of the MTL subcomponents. Location of MTL in the left (**a**) and right hemispheres (**b**) of the whole brain along with a zoomed view (**c**) as well as a tagged zoomed view (**d**). Panels **c** and **d** highlight the various substructures that make up the MTL, posterior parahippocampal gyrus (pPHG), anterior parahippocampal gyrus (aPHG), hippocampal tail (tHi), body (bHi) and head (hHi), as well as medial (mEnt) and lateral (lEnt) entorhinal cortex

To obtain the three subdivisions for the hippocampus we relied on the Hippocampal Subfields and Nuclei of the Amygdala script (v21) with the (0.7 mm) T2w image as the input (Iglesias et al. 2015). Besides segmenting the hippocampus into a set of internal subfields, this script also provides a segmentation of the hippocampus into its head, body and tail sections. In addition, anterior and posterior PHG were obtained by first extracting the parahippocampal gyrus mask from a given participant’s aparc+aseg atlas. Next, to define aPHG and pPHG, we computed an intersection of a plane with the centroid point of the parahippocampal gyrus. The centroid point in the anterior–posterior direction was computed as the mean voxel coordinate of the parahippocampal mask along the y-axis. Consequently, the voxels posterior to this y-plane were defined as pPHG and voxels anterior to the plane as aPHG. To separate the entorhinal cortex in a medial and lateral section we computed its centroid point as the mean coordinate along the *x*-axis and defined medial and lateral sections as above. This, therefore, produced participant-specific MTL subcomponent masks for head, body and tail of the hippocampus, anterior and posterior PHG, and medial and lateral entorhinal cortex (see Fig. 1 for a graphical presentation of the location of the MTL and its various subcomponents for a representative participant).

#### Detection of MTL activation clusters

The next step of the analysis had three goals. First, we attempted to identify those locations of the MTL that were activated during the resting state using a data-driven approach. We first created MTL masks combining bilateral hippocampus, parahippocampal gyrus and entorhinal cortices that were specific to each participant. These masks were similar to those described above in that they were derived from the participant-specific aparc+aseg atlas, but were different because they did not distinguish between the individual MTL subcomponents. This was because we first aimed to detect locations inside the entire MTL that are active during the resting state without taking into account the various MTL subcomponents. To improve accuracy for the group analyses, each participant’s MTL mask was increased in size by one voxel. We then multiplied the masks with the cleaned, whole-brain resting-state fMRI data. This produced 4D fMRI files containing only the timeseries of the voxels within the MTL mask of each participant. We then performed group spatially restricted ICA (group srICA) over these data using FSL melodic (v3.15). Given the smaller sized dataset due to the masking, we used the default method for data reduction (i.e., an initial principal component analysis) by disabling MIGP in the melodic options (Smith et al. 2014).

As mentioned in the Introduction, the main advantage of srICA compared to whole-brain ICA is that a more sensitive decomposition of signals in a specific region can be obtained. As also mentioned above, a well-known problem with fMRI data is that the GRE EPI acquisition protocols are sensitive to local magnetic field inhomogeneities (Devlin et al. 2000) which lead to variability in the tSNR between the different areas of the brain (see Supplementary Fig. S2 for an overview of the tSNR in the current dataset as well as evidence that MTL regions had substantially lower tSNR compared to other areas). A consequence of the multivariate nature of ICA is that signals coming from regions with low tSNR are less likely to be included in a component (connectivity) map. This is because the timecourses of regions with increased noise (i.e., low tSNR) are less likely to correlate with the timecourses of other regions. One way to deal with this issue is to apply ICA to a specific region with a low tSNR. In this case, the ICA procedure will take into account the specific noise profile of that region and enable a more sensitive separation between voxels clusters that correspond to noise and voxel clusters that correspond to signal. In other words, given that the MTL is a region known to have low tSNR (Olman et al. 2009 and see Supplementary Fig. S2), we reasoned that srICA applied to the MTL would lead to a more sensitive detection of signal clusters inside the MTL compared to a whole-brain ICA approach.

One aspect of ICA is that it requires a decision about the number of dimensions under which the analysis is performed. Here, we used a method to determine the optimal number of dimensions for the ICA that was developed in our laboratory (Ezama et al. 2021). Specifically, we tested across a wide range of different dimensions the relationship between a set of components found for a specific dimension and a set of known resting-state networks (Yeo et al. 2011). We then chose the dimension at which this relationship was optimal. Specifically, we first performed ICA on the same dataset at dimensions ranging from 1 to 15 in a stepwise fashion. Next, we obtained whole-brain FC maps derived from all MTL activation clusters for each dimension. These FC maps were obtained using the Dual Regression technique (Nickerson et al. 2017). The dual regression approach consisted of a first regression of the IC outputs of melodic to the cleaned and whole-brain fMRI data for each participant. The output of this first step in dual regression are the time-courses of each independent component for each participant. The second step in dual regression involves a regression of the time-courses associated with each participant-specific IC map against the cleaned fMRI data. This second step produced the whole-brain maps that represent the FC between a specific IC and the rest of the brain.

These whole-brain FC maps obtained for each of the 15 dimensions were then correlated with the 7 resting-state networks of Yeo et al. (2011). This led to 15 correlation matrices of sizes $m \times n$ where *m* refers to the number of dimensions (1–15), and *n* to the number of resting-state networks (here 7). Inspection of these 15 correlation matrices revealed the set of resting-state networks that frequently had high correlation ($r > 0.40$) with a set of components across all dimensions. We then executed an algorithm that found a component if its maximum correlation with a given resting-state network was above a threshold ($r_{\text {max}} > 0.4)$ and if this maximum correlation was sufficiently higher than the second highest correlation ($\frac{r_{\text {max}}}{r_{\text {max}_2}}>1.3$), both within the same IC and within the same resting-state network. On the assumption that a larger number of dimensions leads to more fractionated component clusters, we then chose the smallest dimension at which this algorithm produced the largest number of ICs. This procedure, therefore, detected in a data-driven fashion the optimal number of dimensions for which the srICA produced the largest number of voxel clusters inside the MTL that were both sensitive and specific to known resting-state networks.

#### Group-level analyses of whole-brain FC

To anticipate our results, the previous steps resulted in the detection of a set of FC maps that closely corresponded to a set of known resting-state networks. The next step in the analyses was to assess the statistical reliability of the observed whole-brain maps at the group level. One standard way of performing such an analysis would be to rely on a voxel-based modeling tool such as FSL randomize. However, a general problem with this approach is that it assumes that each voxel represents the exact same brain region across all participants. However, as has been discussed at length elsewhere, there is large variability in brain morphology between participants, and therefore, group-level analyses of this type are sub-optimal (Anticevic et al. 2008; Fischl et al. 2008). Instead, we opted for a different analysis approach that took into account the unique morphology of each participant’s brain. Specifically, we created a dataset that, for each participant, contained average co-activity values for all their cortical and subcortical regions from the aparc+aseg atlas. We obtained these data by intersecting each participant-specific aparc+aseg atlas with each participant-specific whole-brain maps obtained from Dual Regression. We then fitted these data to a linear mixed effect regression model of the form:

1 $$\begin{aligned} Z = \text {hemisphere}\, +\, \text {FC\_map} \,\times \, \text {brain\_region}\, +\, \text {rand (participant)}, \end{aligned}$$

where hemisphere was a discrete co-variable with two levels (left vs right), FC_map was a factor with number of levels equal to the number of ICs corresponding to resting-state networks detected in the previous step, brain_region was a factor with number of levels equal to the sum of the number of cortical and subcortical regions in the aparc+aseg atlas, and participant was a random factor with number of levels equal to the total number of participants (i.e., 172). The dependent variable was the average *Z* value for each brain region computed from the participant-specific Dual Regression maps. Importantly, this mixed-effect model included a random effect term for participant that takes into account the likely between-participant variability that is inherent in these data. In addition, this modeling approach uses participant-specific masks that leads to group-level results that do not violate the assumption of unique brain morphology.

Within this model, our specific interest was in the interaction term of the model ($\text {FC\_map} \times \text {brain\_region}$) that provided a test of the null hypothesis that brain regions would not have differences in mean co-activity values across the different FC maps. In other words, this interaction would not be significant if the different activation clusters detected in the MTL would be connected to the exact same whole-brain resting-state networks. When this interaction was significant, we performed post hoc tests where we compared for each FC map, the average *Z* value for a given region versus the mean of the other regions (an “effect” contrast). This, therefore, produced for each FC map, a list of cortical and subcortical regions from the aparc+aseg atlas that had significantly more co-activity compared to all other regions.

All modeling took place in the statistical computing environment R (v4.0.0). Mixed effect modeling relied on the lme4 package (v1.1.23; Bates et al. 2007). Results from these regression models are presented in the form of ANOVA tables that were computed directly from the output of the mixed effect models using the lmerTest package (v3.1-2; Kuznetsova et al. 2017). *P* values in these models were computed using the Satterthwaite correction for the degrees of freedom. Post hoc testing was performed using the emmeans package (v1.4.6; Lenth et al. 2018) when a given interaction term was significant (i.e., $p < 0.05$). *P* values in these post hoc tests were adjusted for multiple comparisons using the Bonferroni method. We visualized these results using the ggseg (v1.5.4; Mowinckel and Vidal-Piñeiro 2019), and ggpubr packages (v0.3.0; Kassambara 2018).

#### Relative contributions of MTL subcomponents

The previous srICA step provided us with several clusters inside the MTL that co-activated with different resting-state networks. The final step in the analyses was to determine the relative contributions of the seven MTL subcomponents (body hippocampus, anterior PHG, etc.) to the different resting-state networks. To do this, we intersected the participant-specific MTL subcomponent masks (described above) with each participant-specific whole-brain FC map obtained from Dual Regression. These data were then fitted to the same statistical model as described in Equation 1, except that the term brain_region now referred to the seven MTL subcomponents. As before, our specific interest was in the interaction term of the model ($\text {FC\_map} \times \text {brain\_region}$) that provided a test of the null hypothesis of whether the seven MTL subcomponents were activated in the same way across the various FC maps. However, the post hoc tests that were performed when this interaction term was significant differed from those described above. Specifically, to determine the relative contribution of the MTL subcomponents to the different resting-state networks, we first performed pairwise comparisons of all seven MTL subcomponents within each FC map. This produced a list of 21 pairwise comparisons for each FC map with a test statistic (i.e., the *z* ratio, see below) that reflected the degree to which a given MTL subcomponent differed from another MTL subcomponent. These pairwise test statistics were then summed, ordered, and thresholded at > 0. This therefore produced for each detected resting-state network an ordered list of the relative contributions of each MTL subcomponent.

#### Validation analysis

To confirm the reliability of our results, we attempted to validate our findings in a second dataset. This validation dataset consisted of two additional rsfMRI acquisitions from the same participants included in the test dataset. The two rsfMRI scans from the validation set were acquired in a different scanning session (on a different day) as the test set, but relied on the same MRI acquisition parameters. The preprocessing protocol used was the same as described for the test dataset. To validate the results, whole brain FC maps were computed from the data in the validation set using the MTL clusters obtained in the test set. This analysis, therefore, provides a validation of the degree to which the MTL clusters we obtained in the spatially restricted ICA step of the analysis generalize to different datasets. We quantified this step by computing the correlation between the whole-brain FC maps in the test and validation sets, and by comparing the correlations of the whole-brain FC maps with the reference networks of Yeo in the test and validation sets.

## Results

### Detection of MTL activation clusters

The procedure for finding the optimal number of dimensions first returned that across all the 15 dimensions tested, resting-state networks 1 (visual), 2 (somatomotor), 3 (dorsal attention), and 7 (default mode) were most frequently found with correlations $r> 0.40$. In addition, the algorithm found that dimension 7 was the lowest dimension at which the largest number of ICs were strongly and uniquely connected to different resting-state networks. Specifically, we found that for dimension 7, four ICs were strongly and uniquely correlated with four different resting-state networks: IC0 was correlated with the dorsal attention network ($r=0.42$), IC1 was correlated with the somatomotor network ($r=0.53$), IC2 was correlated with the default mode network ($r=0.59$), and IC3 was correlated with the visual network ($r=0.66$; see Table 1 for an overview of the correlations for each IC with all networks). As can be seen in Supplementary Fig. S3, strong correlations ($r > 0.40$) were frequently found for these four networks in other dimensions, suggesting that the detection of these four networks was not idiosyncratic to dimension 7. In addition, as can be seen in Supplementary Fig. S4, we also examined dimensions 20 and 30 and this did not lead to the detection of new networks. Finally, as can be seen in Supplementary Fig. S5, the ICs that were uncorrelated with the reference networks were indeed unlikely to reflect real BOLD signal. Specifically, IC4 seems to reflect signal in CSF, IC5 in draining veins, and IC6 does not reveal much signal in the first place (see also Griffanti et al. 2017, for further information on manually classifying ICs). We can, therefore, conclude that for our data the specific clusters of voxels detected by the ICA using dimension 7 for IC0, IC1, IC2 and IC3 were optimal in connecting with the four known resting-state networks (see also Supplementary Fig. S6 for a direct comparison in overlap between the obtained ICs and Yeo networks).

**Table 1:** Table of correlations of the FC maps with resting-state networks

Yeo et al. (2011), seven networks	IC0	IC1	IC2	IC3
Visual	0.13	0.12	0.02	**0.66**
Somatomotor	0.02	**0.53**	0.03	0.24
Dorsal attention	**0.42**	0.05	0.01	0.16
Ventral attention and salience	0.03	0.01	0.02	0.21
Limbic	0.04	0.10	0.00	0.02
Executive control	0.20	0.02	0.11	0.00
Default mode	0.12	0.20	**0.59**	0.01

Correlations that were strongly and unique correlated with specific resting-state networks are shown in bold

**Fig. 2:**
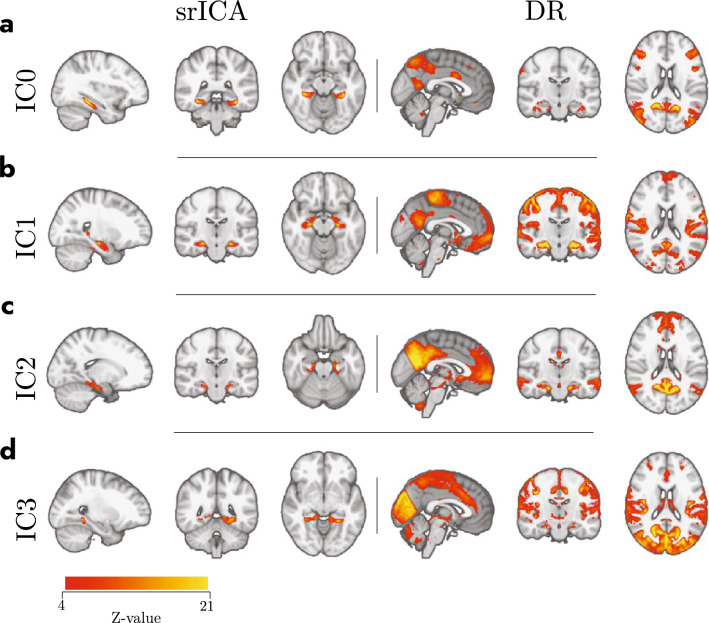
The subset of Independent Components (IC0, first row; IC1, second; IC2, third; IC3, fourth) detected as signal from the spatially restricted ICA (left columns, srICA), and their corresponding large-scale functional connectivity maps derived from Dual Regression (right columns, DR). The color gradient represents the group level of co-activation quantified in *Z* values. Note how different IC hotspots inside the MTL connect to different areas of the brain that highly overlap with resting-state networks (see also Supplementary Fig. S6). *srICA* spatially restricted independent component analysis, *IC* independent component, *DR* dual regression

**Fig. 3:**
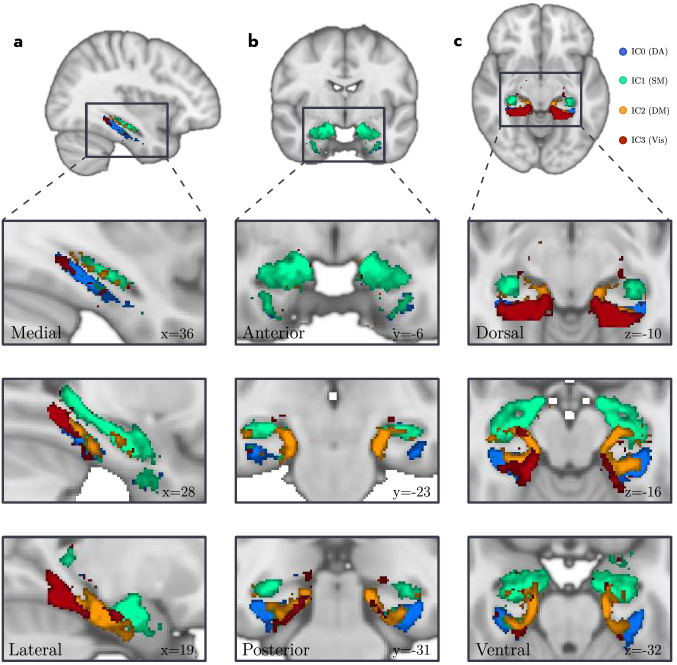
Detailed location of the main target hotspots in the MTL detected with spatially restricted ICA. IC0 (blue), IC1 (green), IC2 (orange) and IC3 (red) in saggital (**a**), coronal (**b**) and axial (**c**) views. Note how ICs both respect and cross anatomical boundaries suggesting inter-structure communication. Slices in 1 mm MNI152 space. *DA* dorsal attention, *SM* somatomotor, *DM* default mode, *Vis* visual

A visual presentation of the precise location of the four ICs along with their whole-brain group-level FC map derived from Dual Regression is presented in Fig. 2. As can be seen in Fig. 2, the four ICs are located at different positions in the MTL and revealed contrasting FC with the rest of the brain, indicating the involvement in different resting-state networks. A more detailed view of the location of each IC within the MTL can be seen in Fig. 3A–C. In this Figure, it can be seen that the four clusters detected by spatially restricted ICA occupy positions within the MTL that both respect and cross anatomical boundaries (e.g., IC0 seems to reflect activity in both parahippocampal gyrus as well as entorhinal cortex). The statistical reliability of the observed whole-brain networks as well as how the detected activation clusters are distributed across the various MTL subcomponents was examined in more detail below.

### Group-level analyses of whole-brain FC

**Fig. 4:**
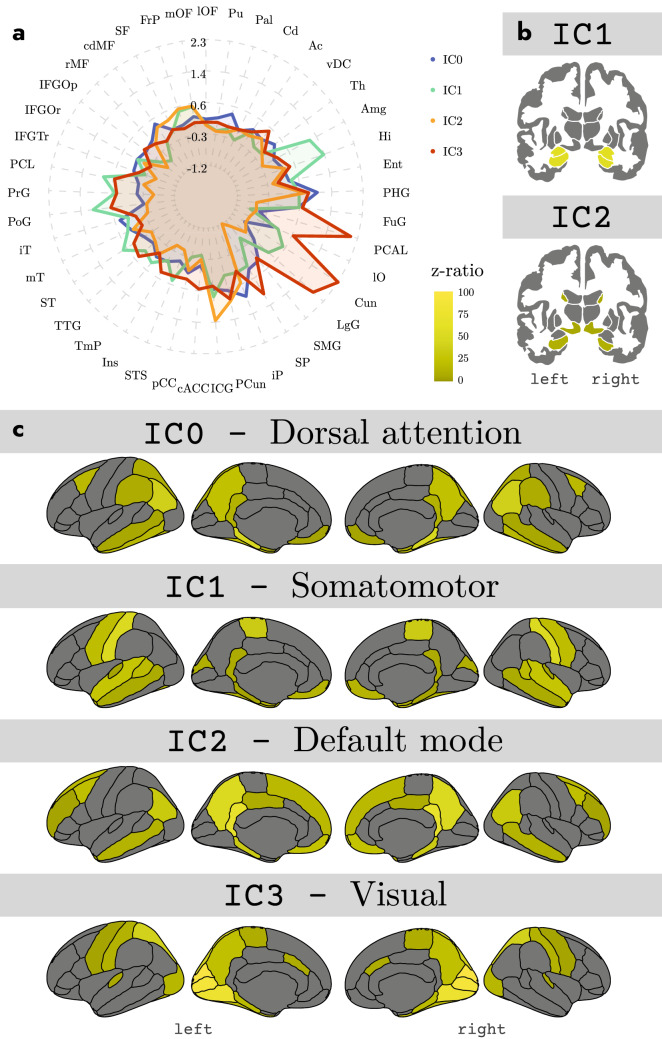
Spiderplot representation of functional connectivity (estimated marginal coefficients) between the four IC hotspots (IC0, IC1, IC2, IC3) and the rest of the brain (**a**), as well as functional connectivity results from group-based analyses in subcortical (**b**) and cortical (**c**) regions for each of the different ICs (see Tables 2, 3, 4 and 5 for details)

Group-level mixed-effect regression analysis revealed main effects of Hemisphere ($F_{1,59508} = 21.72, p < 0.0001$), FC Map ($F_{3,59508} = 2955.38, p < 0.0001$), and Brain Region ($F_{43,59508} = 351.21, p < 0.0001$). Most relevant for our present purposes, there was a significant interaction between FC Map and Brain Region ($F_{129,59508} = 589.18, p < 0.0001$), suggesting that co-activity values of brain regions differed between the various whole-brain FC maps associated with each IC. As can be seen in Table 2, post hoc analyses revealed that IC0 and its corresponding FC map revealed regions typically associated with the dorsal-attention network like the inferior and superior parietal cortex, as well as lateral frontal areas. Similarly, as can be seen in Table 3, IC1 and its associated FC map showed regions typically associated with the somatomotor network like the amygdala and post- and pre-central gyri. In addition, as can been seen in Table 4, IC2 and its corresponding FC map revealed regions generally found in the default mode network, like the isthmus cingulate (retrosplenial cortex), the precuneus, and the medial orbitofrontal cortex. Finally, as can be seen in Table 5, IC3 and its associated FC map revealed regions associated with the visual network like the cuneus and pericalcarine sulcus (see also Fig. 4B and C for a visual presentation of these results).

**Table 2:** Cortical and subcortical areas showing reliable co-activity with IC0 (correlated with Dorsal Attention network) relative to the mean co-activity value of all other areas

Region	*z* ratio	*p* value
Parahippocampal	47.72	~0.000E+00
Inferior parietal	40.13	~0.000E+00
Precuneus	24.03	5.577E–126
Fusiform	20.59	1.569E–92
Caudal middle frontal	17.42	2.406E–66
Inferior temporal	16.61	2.535E–60
Isthmus cingulate	11.68	7.301E–30
Superior parietal	9.17	2.068E–18
Supramarginal	8.38	2.194E–15
Entorhinal	7.79	2.789E–13
Middle temporal	5.78	3.301E–07
Medial orbitofrontal	4.10	1.802E–03

*p* values corrected for multiple comparisons using Bonferroni correction

### Relative contributions of MTL subcomponents

**Fig. 5:**
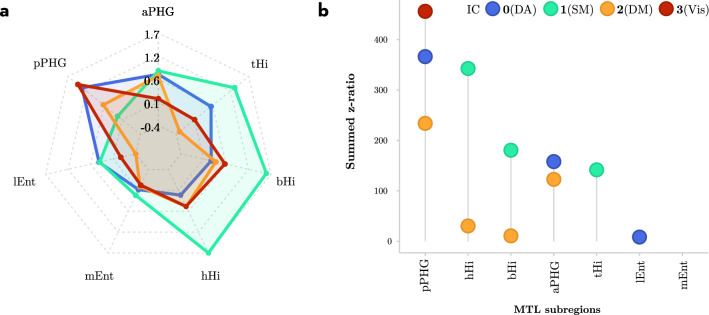
Spiderplot representation of co-activity values (estimated marginal coefficients) within the 7 MTL regions for each of the four ICs (**a**), as well as the relative contribution (in terms of summed z-values) from each MTL region to each of the four ICs (**b**). Note that, for example, IC0 (Dorsal Attention, blue dots) relies on contributions from pPHG and aPHG and lateral Ent, and that IC2 (Default Mode, green dots) relied primarily on pPHG, aPHG, and on head and body of the hippocampus. *DA* dorsal attention, *SM* somatomotor, *DM* default mode, *Vis* visual

Group-level mixed-effect regression analyses that examined the relative contribution of the MTL subcomponents to each resting-state network revealed main effects of Hemisphere ($F_{1,9432} = 45.96, p < 0.0001$), Brain Region ($F_{6,9432} = 1867.63, p < 0.0001$) and FC Map ($F_{3,9432} = 2378.99, p < 0.0001$). Again, important for our present purposes, the interaction between Region and FC Map was highly significant ($F_{18,9432} = 1442.85, p < 0.0001$), suggesting that average co-activity values for each MTL subcomponent differed between the four ICs. Further exploration of this interaction using pairwise comparisons of the seven MTL subcomponents within each IC and then ranking the summed *z* ratios revealed the relative contributions of each MTL subcomponent. Specifically, as can be seen in Table 6, summed *z* ratios in IC0 (correlated with the dorsal-attention network) were strongest in pPHG, then in aPHG and finally in lEnt. Similarly, Table 6 showed that for IC1 (correlated with the somatomotor network), summed *z* ratios were strongest in hHi followed by bHi, and tHi. In addition, Table 6 showed that for IC2 (correlated with the default mode network) summed *z* ratios in descending order were ranked pPHG, aPHG, hHi and bHi. Finally, Table 6 showed that for IC3, summed *z* ratios were strongest in pPHG (see also Figure 5 for a graphical presentation of these results).

**Table 3:** Cortical and subcortical areas showing reliable co-activity with IC1 (correlated with Somatomotor network) relative to the mean co-activity value of all other areas

Region	*z* ratio	*p* value
Amygdala	55.30	~0.000E+00
Hippocampus	53.71	~0.000E+00
Postcentral	49.85	~0.000E+00
Paracentral	34.58	2.413E–260
Superior temporal	27.76	5.009E–168
Precentral	21.26	1.237E–98
Bankssts	19.23	8.143E–81
Medial orbitofrontal	13.27	1.406E–38
Entorhinal	12.17	1.874E–32
Fusiform	11.51	5.215E–29
Temporal pole	10.15	1.421E–22
Cuneus	10.00	6.511E–22
Frontal pole	8.82	4.865E–17
Isthmus cingulate	7.39	6.378E–12
Middle temporal	7.19	2.866E–11
Transverse temporal	3.83	5.402E–03
Parahippocampal	3.62	1.279E–02

*p* values corrected for multiple comparisons using Bonferroni correction

### Validation results

Calculation of the whole-brain FC maps in the validation dataset using the MTL clusters obtained from the test set described above showed highly similar results to those obtained in the test set (see Figure S7). Correlation of FC maps in the test and validation sets showed highly reliable correlations. Specifically, the FC maps associated with IC0 (DA), IC1 (SM), IC2 (DMN) and IC3 (VIS), correlated $r=0.95, r=0.96, r=0.98, r=0.97$ between the test and validation set, respectively. Finally, the pattern of correlations between the obtained FC maps in the validation set and the Yeo reference networks was highly similar (see Table S1). We, therefore, conclude the MTL clusters that we report are robust and generalize to different datasets.

**Table 4:** Cortical and subcortical areas showing reliable co-activity with IC2 (correlated with Default Mode network) relative to the mean co-activity value of all other areas

Region	*z* ratio	*p* value
Isthmus cingulate	70.52	~0.000E+00
Precuneus	50.65	~0.000E+00
Parahippocampal	34.93	1.301E–265
Inferior parietal	32.82	1.256E–234
Rostral anterior cingulate	32.67	1.781E–232
Frontal pole	26.58	5.334E–154
Medial orbitofrontal	26.31	7.064E–151
Caudal middle frontal	21.98	1.828E–105
Superior frontal	18.42	3.889E–74
Middle temporal	13.09	1.656E–37
Hippocampus	11.37	2.489E–28
Posterior cingulate	8.69	1.499E–16
Caudate	7.09	5.923E–11
Ventral DC	6.21	2.345E–08
Temporal pole	4.91	3.874E–05
Rostral middle frontal	4.19	1.188E–03
Brain stem	3.53	1.777E–02

*p* values corrected for multiple comparisons using Bonferroni correction

### Complementary results

One concern with the results reported above is that we only compared the whole-brain FC maps with the set of 7 reference networks of Yeo et al. (2011). In complementary analyses, we examined whether our results would generalize to the 17 reference networks of Yeo et al. (2011), the 10 networks in Smith et al. (2009) and the 18 networks in Allen et al. (2011). Specifically, we correlated the whole brain FC maps that we obtained for dimension 7 with these additional reference networks. The results from these correlations are shown in Supplementary Tables S2–4. There are two noteworthy observations from these results. First, the results we obtained with the 17 networks of Yeo et al. (2011) and the 10 networks of Smith et al. (2009) were comparable to those obtained above. Specifically, IC3 yielded the highest correlation with the visual network, IC2 with the default mode, and IC1 with the somatomotor network for both the 17 reference networks of Yeo et al. (2011) and the 10 networks in Smith et al. (2009). However, IC0 (associated with dorsal attention in the set of 7 networks) correlated $r=0.28$ the default mode network C and $r=0.20$ with the dorsal attention network A in the 17 networks of Yeo et al. (2011), and $r=0.29$ with the frontoparietal network in the set of Smith et al. (2009). This variability likely reflects a further splintering of networks and label differences between the reference sets.

Second, the correlations between our obtained results and the 18 networks of Allen et al. (2011) resulted in generally low correlations (see Supplementary Table S4). Although we cannot provide a clear explanation of these low correlations at this point, we note that these results are not in line with these observed with the other reference sets and that they resemble the low correlations found by Ruiz-Rizzo et al. (2020) that also used this reference set. We discuss this issue in more detail below.

**Table 5:** Cortical and subcortical areas showing reliable co-activity with IC3 (correlated with Visual network) relative to the mean co-activity value of all other areas

Region	*z* ratio	*p* value
Cuneus	99.93	~0.000E+00
Pericalcarine	89.54	~0.000E+00
Lingual	77.22	~0.000E+00
Superior parietal	40.98	~0.000E+00
Precuneus	23.29	2.205E–118
Parahippocampal	18.32	2.276E–73
Fusiform	16.28	5.737E–58
Lateral occipital	15.69	7.799E–54
Postcentral	11.46	9.415E–29
Paracentral	8.86	3.329E–17
Caudal anterior cingulate	7.90	1.164E–13
Transverse temporal	6.98	1.274E–10
Precentral	4.16	1.375E–03

*p* values corrected for multiple comparisons using Bonferroni correction

**Table 6:** Strongest contrast for MTL substructures for each IC

Region	IC	Summed *z* ratio	Rank	Network
pPHG	0	366.37	1	
aPHG	0	158.15	2	Dorsal attention
lEnt	0	8.44	3	
hHi	1	342.85	1	
bHi	1	180.64	2	Somatomotor
tHi	1	141.79	3	
pPHG	2	233.97	1	
aPHG	2	122.92	2	Default mode
hHi	2	30.40	3	
bHi	2	10.85	4	
pPHG	3	456.13	1	Visual

Contrast based on the sum of pairwise *z* ratio differences for all substructures. Rank indicates the order of the substructures relative contribution for each IC

## Discussion

The aim of the current study was to characterize the different resting-state networks that co-activate with the MTL as well as detail how the different MTL subcomponents contribute to these resting-state networks. We examined this issue using the high spatial resolution 7T rsfMRI dataset from the HCP with a data-driven method that applied ICA in a manner that was restricted to the MTL. We found that during the resting state, our method detected four activation clusters that were spread across the various subcomponents of the MTL. Using Dual Regression and mixed effect regression techniques to estimate reliability across participants, we found that these four activation clusters were functionally connected to four different whole-brain resting-state networks that relied on different contributions of MTL subcomponents. Specifically, we found that the dorsal attention network (detected with $r = 0.42$) relied primarily on the parahippocampal gyrus and entorhinal cortex, the somatomotor network ($r = 0.53$) on the hippocampus, the default mode network ($r = 0.59$) on both parahippocampal gyrus and hippocampus, and the visual network ($r = 0.66$) on the parahippocampal cortex (see Table 6 for details). These results were validated with high replication ($r>0.95$) in a separate dataset.

Previous studies have reported inconsistent results on the number of whole-brain networks that co-activate with the MTL. Whereas, the classical view is that the MTL is connected to a posterior and an anterior network (Kahn et al. 2008; Libby et al. 2012; Qin et al. 2016; Ranganath and Ritchey 2012; Ritchey et al. 2015; Schröder et al. 2015), more recent studies have found that the MTL relies on additional networks beyond these two traditionally proposed (Ruiz-Rizzo et al. 2020; Wang et al. 2016; Plachti et al. 2019). The current results are in line with these more recent studies in that they show that the MTL is connected to additional resting-state networks. Specifically, the current study shows that the MTL was connected to the dorsal attention, somatomotor, default mode and visual networks (see Table 1 for details). Three of these four networks have a clear correspondence to the posterior and anterior networks previously identified. Specifically, the default mode and visual network likely reflect the previously identified posterior network, while the somatomotor network likely reflects the anterior network. This interpretation rests on the specific overlap of regions usually associated with the posterior/anterior networks and with the regions found to be linked to the resting-state networks obtained in our study. In particular, the posterior network is typically associated with posterior regions like the retrosplenial cortex, precuneus (Ranganath and Ritchey 2012; Ritchey et al. 2015) as well as with occipital areas (Libby et al. 2012; Wang et al. 2016). As can be seen in Tables 4 and 5 these regions were exactly among those with the highest co-activation in the set of regions linked to the visual and default mode networks found in our study. Similarly, the anterior network is typically associated with the amygdala and orbitofrontal cortex (Ranganath and Ritchey 2012; Ritchey et al. 2015), and as can be seen in Table 3, these regions also appear among the most co-activated in the set of regions associated with the somatomotor network. The current results, therefore, suggest that, in a resting-state context, the posterior and anterior networks typically linked with the MTL are the visual, default mode and somatomotor networks, respectively.

Although the current results are in line with those studies that have argued for a more expanded connectivity between the MTL and the rest of the brain (Ruiz-Rizzo et al. 2020; Wang et al. 2016; Plachti et al. 2019), they differ from these previous studies in some details. First, as mentioned in the Introduction, a recent study by Ruiz-Rizzo et al. (2020) concluded that MTL was connected with five different resting-state networks. Using the reference set of 20 networks of Allen et al. (2011), they found that MTL connected with default mode, salience, frontal, basal ganglia and visual networks. One general problem with this study are the relatively low correlations between the observed and reference networks (i.e., visual, frontal and salience networks all had $r < 0.15$). Indeed, the problem here may to be related to the specific reference atlas because when we used the Allen et al. (2011) atlas as a reference, correlations between obtained networks and the reference set were also relatively low (see Complementary Results and Supplementary Table S4). Given that correlations were substantially higher in the reference sets of Yeo et al. (2011) and Smith et al. (2009), future studies should be geared towards resolving these rather remarkable discrepancies between sets of reference networks. In addition, a study by Plachti et al. (2019) found that during the resting state, functional connectivity between the hippocampus and the rest of the brain could be described by 3, 5 or even 7 clusters. This seems at odds with our observation that the hippocampus was involved in only two networks (default mode and somatomotor networks; see also Ezama et al. 2021). However, this interpretation is complicated by the fact that Plachti et al. (2019) focused on the internal parcellation of the hippocampus and did not present the whole-brain functional connectivity of the obtained parcellations. Future studies that employ the consensus clustering technique used by Plachti et al. (2019) while also reporting whole-brain connectivity maps should be able to resolve this issue. In short, although the current results as well as the set of studies discussed here support the notion of expanded connectivity of the MTL, specific details regarding the influence of the particular reference set and pattern of whole-brain connectivity remain to be resolved.

The current results also provide insight into how the various MTL subcomponents contribute to these four resting-state networks. Specifically, the visual network relied primarily on posterior sections of the parahippocampal gyrus (PHG), and the dorsal attention network primarily on a posterior-to-anterior gradient along the parahippocampal long-axis and lateral entorhinal cortex (pPHG–aPHG–lEnt, in order of relative contribution). In addition, the default mode network relied on a more complex pattern of co-activation in both parahippocampal gyrus and hippocampus with opposite gradients in these two structures: In the parahippocampal gyrus the gradient was in the posterior–anterior (pPHG–aPHG) direction; whereas in hippocampus, it was in the anterior–posterior (head–body of hippocampus) direction. Finally, the somatomotor network relied primarily on the hippocampus with an anterior–posterior gradient (head–body–tail of hippocampus; see Table 6, and Figure 5 for details). These results are generally consistent with those previously observed. Specifically, the posterior network has traditionally been associated with pPHG and middle to posterior sections of the hippocampus (Kahn et al. 2008; Libby et al. 2012; Qin et al. 2016; Ranganath and Ritchey 2012; Ritchey et al. 2015; Ruiz-Rizzo et al. 2020; Schröder et al. 2015; Wang et al. 2016). This is in line with our observations that the visual and default mode networks strongly co-activate with posterior sections of the parahippocampal gyrus and hippocampus. In addition, the anterior network is traditionally associated with anterior sections of the parahippocampal gyrus (aPHG, including perirhinal cortex) and anterior sections of the hippocampus. Interestingly, our results revealed that the somatomotor network, that included areas typically associated with the anterior network like the amygdala and medial orbitofrontal cortex, relied primarily on the hippocampus and not on the parahippocampal gyrus (see Table 3 and Figure 3). One possible explanation for this is that the anterior network previously observed in low spatial resolution data represented a mix between the somatomotor and dorsal attention networks thereby explaining the involvement of the aPHG. In sum, the current results, therefore, suggest that MTL plays a role in four different resting-state networks where these networks display distinct configurations of MTL subcomponents.

Although the MTL is traditionally linked with episodic memory (e.g., Squire & Zola-Morgan 1991), more recent studies have shown the involvement of this structure in a wide range of cognitive functions such as short-term memory (Ranganath and Blumenfeld 2005), visual perception (Barense et al. 2012), attention (Aly and Turk-Browne 2016; Córdova et al. 2019; Ruiz et al. 2020), and language and conceptual processing (Duff and Brown-Schmidt 2012; Mack et al. 2016; Piai et al. 2016). For example, a recent study by Ruiz et al. (2020) revealed that patients with MTL lesions showed impaired performance in an attention task that relied on visual perception suggesting that the MTL plays a role in attention processes. The results reported here are in line with these studies in that they highlight the variety of resting-state networks to which the MTL contributes. Specifically, although resting-state studies can only make limited claims about function, our observation of the MTL in visual and dorsal attention networks seem to be in line with previous studies that have emphasized the implication of MTL in perception and attentional processes (Aly and Turk-Browne 2016; Córdova et al. 2019; Ruiz et al. 2020). The current observation that the MTL is involved in four different resting-state networks, therefore, further underscores the notion that the MTL may be involved in a more abstract type of processing (e.g., relational) that plays an important part in many different cognitive domains.

Our study has several limitations. First, our conclusion that MTL relies on four particular resting-state networks is based on the specific reference atlas of Yeo et al. (2011). However, as we show in Supplementary Tables S2–4, correlations with the 17 network atlas of Yeo et al. (2011) and the 10 networks of Smith et al. (2009) yielded highly similar results. In addition, group-level FC analysis relied on averages across voxels in a brain region. An obvious disadvantage in this approach is that there are limitations on the spatial precision with which an effect may be localized. We think such concerns may be further mitigated by using procedures that segment brain regions to more fine grained parcels (e.g., Schaefer et al. 2018). Finally, although we compared the contributions of 7 different MTL subcomponents to whole-brain resting-state networks, we did not explicitly include the perirhinal cortex. The reason for this is twofold. First, the perirhinal cortex simply does not form part of the standard Desikan–Killany atlas that we used for our segmentations (Desikan et al. 2006). Second, the precise anatomical definition of perirhinal cortex remains highly disputed (Suzuki and Amaral 1994; Augustinack et al. 2014), which complicates an accurate automatic segmentation. Thus, although the usefulness of this structure is clear (Augustinack et al. 2014; Libby et al. 2012), the integration of this structure into an automatic segmentation pipeline that also includes other MTL structures has prevented us from analyzing the contribution of this structure at this moment in time.

To conclude, the current study used a high spatial resolution dataset to examine the different whole-brain resting-state networks that co-activate with the MTL. A secondary goal was to examine how the different MTL subcomponents contribute to these resting-state networks. We found that the MTL co-activates with the default mode, somatomotor, visual and dorsal attention networks. Our results revealed that these networks are subserved by distinct configurations of MTL subcomponent co-activity, where the default mode network relied on a combination of activity in parahippocampal gyrus and hippocampus, the somatomotor network on the hippocampus, the visual network on the parahippocampal gyrus, and the dorsal attention network on the parahippocampal gyrus and the entorhinal cortex. These results go beyond previous studies that have associated MTL with a posterior and an anterior network (Kahn et al. 2008; Libby et al. 2012; Qin et al. 2016; Ranganath and Ritchey 2012; Ritchey et al. 2015; Ruiz-Rizzo et al. 2020; Schröder et al. 2015; Wang et al. 2016; Barnett et al. 2019; Vincent et al. 2006). They are in line with previous FC studies that have found that the MTL is linked to additional networks (Ruiz-Rizzo et al. 2020; Wang et al. 2016; Plachti et al. 2019) and are suggestive of a functional role of MTL that goes beyond episodic memory (Aly and Turk-Browne 2016; Barense et al. 2012; Córdova et al. 2019; Duff and Brown-Schmidt 2012; Ranganath and Blumenfeld 2005; Ruiz et al. 2020). Finally, the current results are obtained from young healthy adults and therefore establish a baseline pattern of how various MTL subcomponents contribute to known resting-state networks. In the future, we hope to examine how these patterns change with aging and pathology.

## Supplementary Information

Below is the link to the electronic supplementary material.

Supplementary file 1 (pdf 4139 KB)

## Data Availability

Data were provided by the Human Connectome Project, WU-Minn Consortium (Principal Investigators: David Van Essen and Kamil Ugurbil; 1U54MH091657) funded by the 16 NIH Institutes and Centers that support the NIH Blueprint for Neuroscience Research; and by the McDonnell Center for Systems Neuroscience at Washington University. The data that support the findings of this study are openly available from Human Connectome Project (www.humanconnectome.org).

## Abbreviations

MTL: Medial temporal lobe
DMN: Default mode network
rfMRI: Resting-state functional magnetic resonance imaging
MNI: Montreal Neurological Institute 152
aPHG: Anterior parahippocampal cortex
pPHG: Posterior parahippocampal cortex
lEnt: Lateral entorhinal cortex
mEnt: Medial entorhinal cortex
hHi: Head of the hippocampus
bHi: Body of the hippocampus
tHi: Tail of the hippocampus
Th: Thalamus proper
Cd: Caudate
Pu: Putamen
Pal: Pallidum
Hi: Hippocampus
Amg: Amygdala
Ac: Accumbens area
vDC: Ventral DC
STS: Bankssts
cACC: Caudal anterior cingulate
cdMF: Caudal middle frontal
Cun: Cuneus
Ent: Entorhinal
FuG: Fusiform
iP: Inferior parietal
iT: Inferior temporal
ICG: Isthmus cingulate
lO: Lateral occipital
lOF: Lateral orbitofrontal
LgG: Lingual gyrus
mOF: Medial orbitofrontal
mT: Middle temporal
PHG: Parahippocampal gyrus
PCL: Paracentral
IFGOp: Pars opercularis
IFGOr: Pars orbitalis
IFGTr: Pars triangularis
PCAL: Pericalcarine
PoG: Postcentral
pCC: Posterior cingulate
PrG: Precentral
PCun: Precuneus
rACg: Rostral anterior cingulate
rMF: Rostral middle frontal
SF: Superior frontal
SP: Superior parietal
ST: Superior temporal
SMG: Supramarginal
FrP: Frontal pole
TmP: Temporal pole
TTG: Transverse temporal
Ins: Insula

## Code availability

Code will be made available upon reasonable request.
