# Supplementary material for: Medial temporal lobe contributions to resting-state networks
Source: Brain Struct Funct. 2022 Jan 18;227(3):995–1012. doi: 10.1007/s00429-021-02442-1 (PMC8930967; doi:10.1007/s00429-021-02442-1)
Supplement: Supplementary file 1 — (pdf 4139 KB) [file 429_2021_2442_MOESM1_ESM.pdf]

# Medial temporal lobe contributions to resting-state networks

Sara Seoane[1,3,4], Cristián Modroño[2,3,4], José Luis González Mora[2,3,4] and Niels Janssen[1,3,4]

[1]Faculty of Psychology, University of La Laguna, Spain

[2]Faculty of Health Sciences, University of La Laguna, Spain

[3]Institute of Biomedical Technologies, University of La Laguna, Spain

[4]Institute of Neurosciences, University of La Laguna, Spain

Corresponding Author:

Niels Janssen

Tel: +34 922317502

e-mail: njanssen@ull.es

# Tables

Table 1: Table of correlations of the FC maps with resting-state networks in the validation set.

| Yeo et al. (2011), 7 networks | IC0 | IC1 | IC2 | IC3 |
|---|---|---|---|---|
| Visual | 0.13 | 0.14 | 0.02 | 0.65 |
| Somatomotor | 0.02 | 0.47 | 0.03 | 0.25 |
| Dorsal Attention | 0.46 | 0.01 | 0.01 | 0.15 |
| Ventral Attention & Salience | 0.05 | 0.01 | 0.02 | 0.22 |
| Limbic | 0.04 | 0.13 | 0.01 | 0.02 |
| Executive Control | 0.21 | 0.02 | 0.12 | 0.00 |
| Default Mode | 0.11 | 0.25 | 0.61 | 0.01 |

Table 2: Table of correlations of the FC maps with resting-state networks for 17 dimensions

| | Correlation with FC maps | | | | |
|---|---|---|---|---|---|
| Yeo et al. (2011), 17 networks | | IC0 | IC1 | IC2 | IC3 |
| Visual | 1 - central | 0.01 | 0.07 | 0.01 | 0.19 |
| | 2 - peripheral | 0.01 | 0.06 | 0.01 | 0.36 |
| Somatomotor | 3 - somatomotor A | 0.01 | 0.33 | 0.01 | 0.12 |
| | 4 - somatomotor B | 0.01 | 0.18 | 0.01 | 0.12 |
| Dorsal attention | 5 - dorsal attention A | 0.20 | 0.04 | 0.01 | 0.15 |
| | 6 - dorsal attention B | 0.07 | 0.06 | 0.01 | 0.07 |
| Ventral attention and salience | 7 - salience/ventral attention A | 0.01 | 0.01 | 0.01 | 0.15 |
| | 8 - salience/ventral attention B | 0.00 | 0.01 | 0.00 | 0.07 |
| Limbic | 9 - limbic A | 0.05 | 0.08 | 0.01 | 0.01 |
| | 10 - limbic B | 0.01 | 0.01 | 0.01 | 0.01 |
| Executive control | 11 - control A | 0.21 | 0.01 | 0.00 | 0.00 |
| | 12 - control B | 0.02 | 0.01 | 0.13 | 0.02 |
| | 13 - control C | 0.08 | 0.01 | 0.11 | 0.07 |
| Default mode | 14 - default A | 0.08 | 0.12 | 0.47 | 0.01 |
| | 15 - default B | 0.01 | 0.11 | 0.14 | 0.02 |
| | 16 - default C | 0.28 | 0.04 | 0.19 | 0.05 |
| | 17 - Temporal parietal | 0.00 | 0.11 | 0.01 | 0.05 |

Note: correlations reported in this table were calculated using `fslcc` on the FC maps thresholded at 2.

Table 3: Table of correlations of the FC maps with Smith et al. (2009) resting-state networks

| Correlation with FC maps | | | | |
|---|---|---|---|---|
| Smith et al. (2009), 10 Networks | IC0 | IC1 | IC2 | IC3 |
| 1 - Visual (medial) | 0.07 | 0.00 | 0.02 | 0.55 |
| 2 - Visual (occipital pole) | 0.01 | 0.01 | 0.04 | 0.08 |
| 3 - Visual (lateral visual) | 0.15 | 0.19 | 0.04 | 0.31 |
| 4 - Default mode | 0.13 | 0.23 | 0.63 | 0.09 |
| 5 - Cerebellum | 0.08 | 0.03 | 0.05 | 0.02 |
| 6 - Sensorimotor | 0.04 | 0.31 | 0.00 | 0.19 |
| 7 - Auditory | 0.00 | 0.23 | 0.07 | 0.18 |
| 8 - Executive control | 0.00 | 0.02 | 0.16 | 0.13 |
| 9 - Frontoparietal (right) | 0.11 | 0.01 | 0.18 | 0.06 |
| 10 - Frontoparietal (left) | 0.29 | 0.05 | 0.14 | 0.01 |

Note: correlations reported in this table were calculated using `fslcc` on the FC maps thresholded at 2.

Table 4: Table of correlations of the FC maps with Allen et al. (2011) resting-state networks

| | Correlation with FC maps | | | |
|---|---|---|---|---|
| Allen et al. (2011), 28 Networks | IC0 | IC1 | IC2 | IC3 |
| 1 - Sensorimotor | 0.04 | 0.11 | 0.01 | 0.12 |
| 2 - Auditory | 0.16 | 0.04 | 0.06 | 0.02 |
| 3 - Frontal | 0.00 | 0.05 | 0.00 | 0.02 |
| 4 - Basal ganglia | 0.07 | 0.15 | 0.08 | 0.03 |
| 5 - Sensorimotor | 0.05 | 0.01 | 0.09 | 0.03 |
| 6 - Sensorimotor | 0.05 | 0.02 | 0.04 | 0.03 |
| 7 - Default Mode | 0.00 | 0.02 | 0.10 | 0.07 |
| 8 - Sensorimotor | 0.11 | 0.03 | 0.19 | 0.06 |
| 9 - Attention | 0.09 | 0.04 | 0.12 | 0.15 |
| 10 - Sensorimotor | 0.01 | 0.04 | 0.07 | 0.01 |
| 11 - Visual | 0.03 | 0.02 | 0.01 | 0.02 |
| 12 - Frontal | 0.05 | 0.08 | 0.02 | 0.04 |
| 13 - Visual | 0.03 | 0.01 | 0.03 | 0.04 |
| 14 - Frontal | 0.02 | 0.00 | 0.01 | 0.04 |
| 15 - Visual | 0.00 | 0.04 | 0.01 | 0.04 |
| 16 - Frontal | 0.03 | 0.03 | 0.01 | 0.05 |
| 17 - Default Mode | 0.06 | 0.06 | 0.08 | 0.28 |
| 18 - Attention | 0.06 | 0.03 | 0.10 | 0.19 |
| 19 - Default Mode | 0.04 | 0.08 | 0.05 | 0.36 |
| 20 - Attention | 0.04 | 0.10 | 0.04 | 0.07 |
| 21 - Sensorimotor | 0.02 | 0.08 | 0.04 | 0.09 |
| 22 - Visual | 0.04 | 0.01 | 0.02 | 0.06 |
| 23 - Attention | 0.11 | 0.05 | 0.05 | 0.09 |
| 24 - Visual | 0.01 | 0.01 | 0.01 | 0.15 |
| 25 - Visual | 0.01 | 0.00 | 0.03 | 0.01 |
| 26 - Default Mode | 0.09 | 0.06 | 0.08 | 0.12 |
| 27 - Attention | 0.06 | 0.04 | 0.02 | 0.01 |
| 28 - Attention | 0.15 | 0.03 | 0.22 | 0.16 |

Note: correlations reported in this table were calculated using `fslcc` on the FC maps thresholded at 2.

# Figures

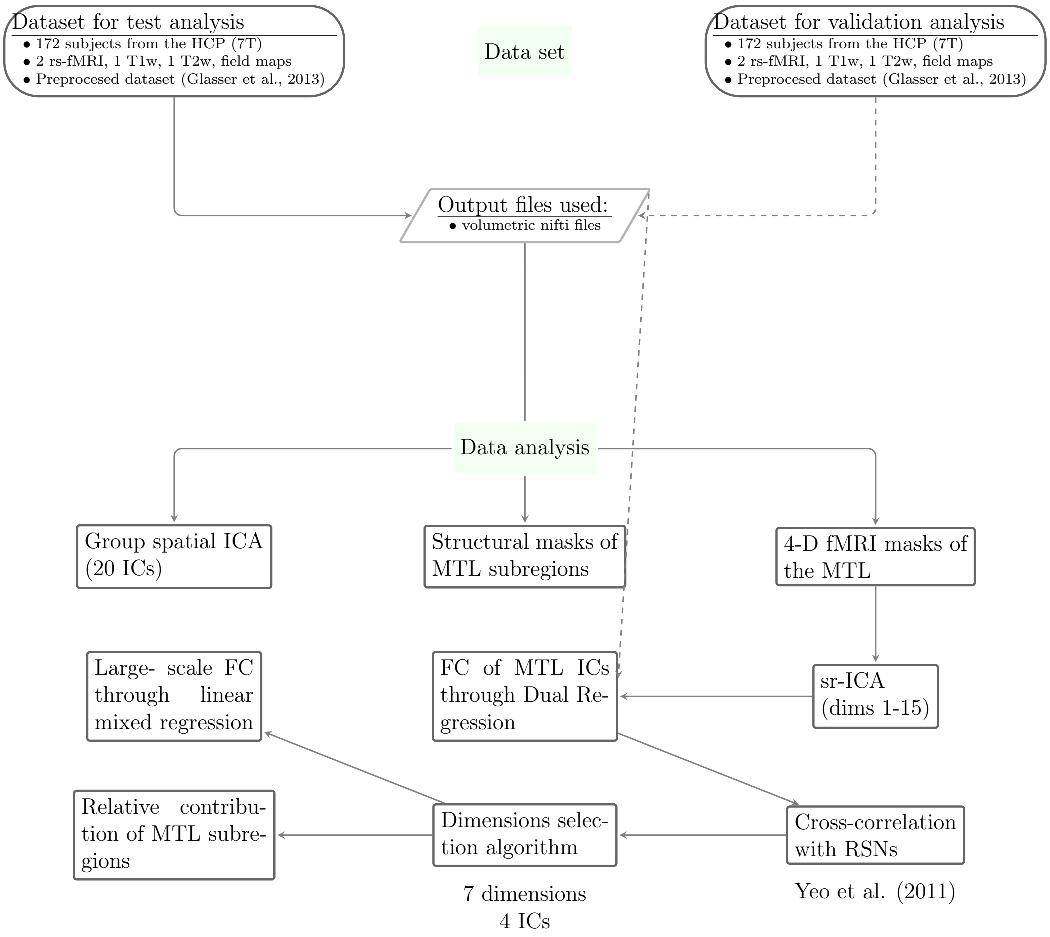


Figure 1: Chart of the data analysis flow. Abbreviations: FC, Functional Connectivity; HCP, Human Connectome Project; IC, Independent Component; ICA, Independent Component Analysis; MTL, Medial Temporal Lobe; RSN, resting-state networks.

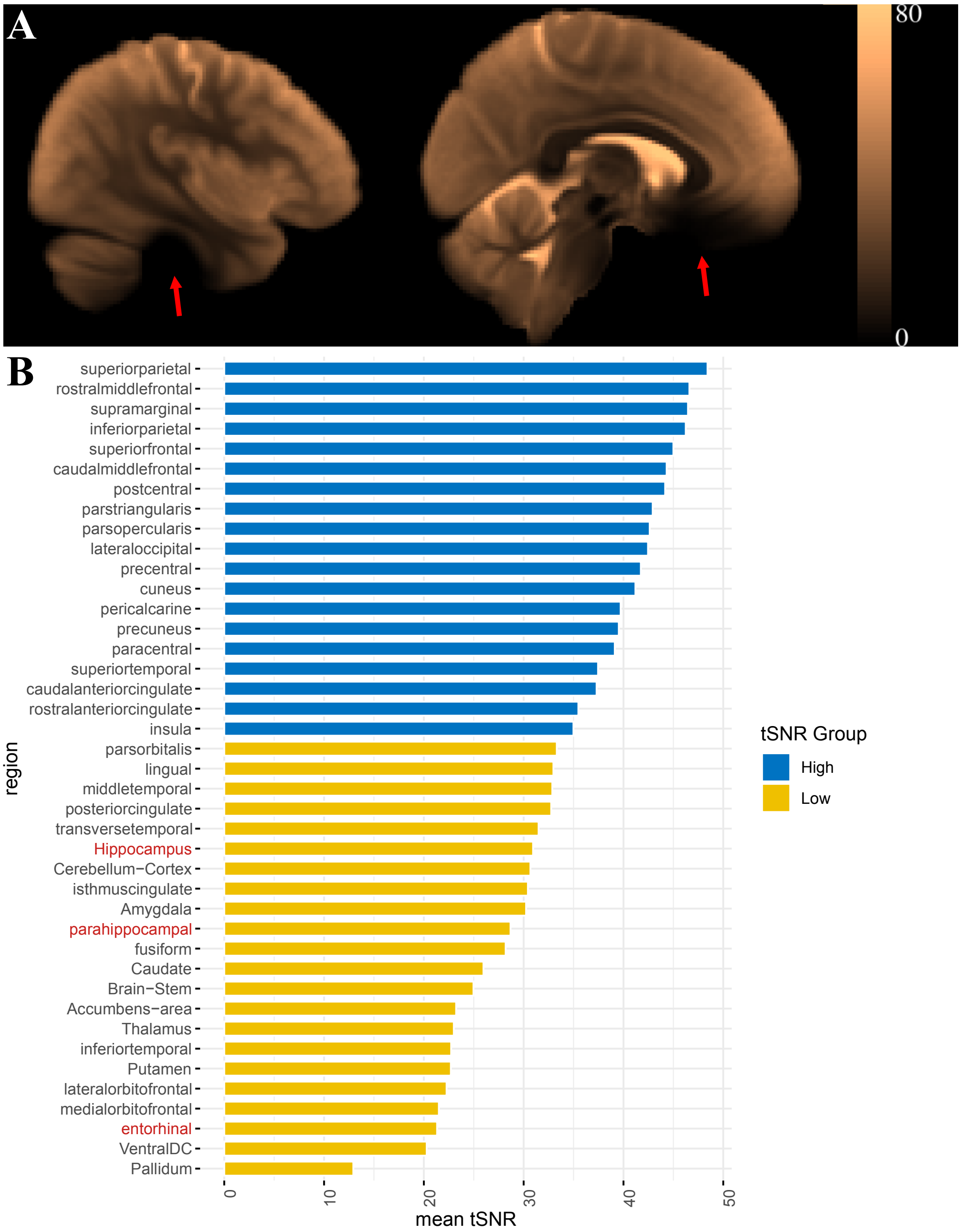


Figure 2: Mean voxel-wise tSNR computed from the first two resting-state datasets (with opposite phase encoding) across all participants (A) and mean tSNR per region split into Low (below mean) or High (above mean) groups (B). Note typical signal dropout in inferior temporal and frontal regions (red arrows), large variability in tSNR across brain regions, and below mean tSNR in regions that comprise the MTL (hippocampus, parahippocampal gyrus and entorhinal cortex).

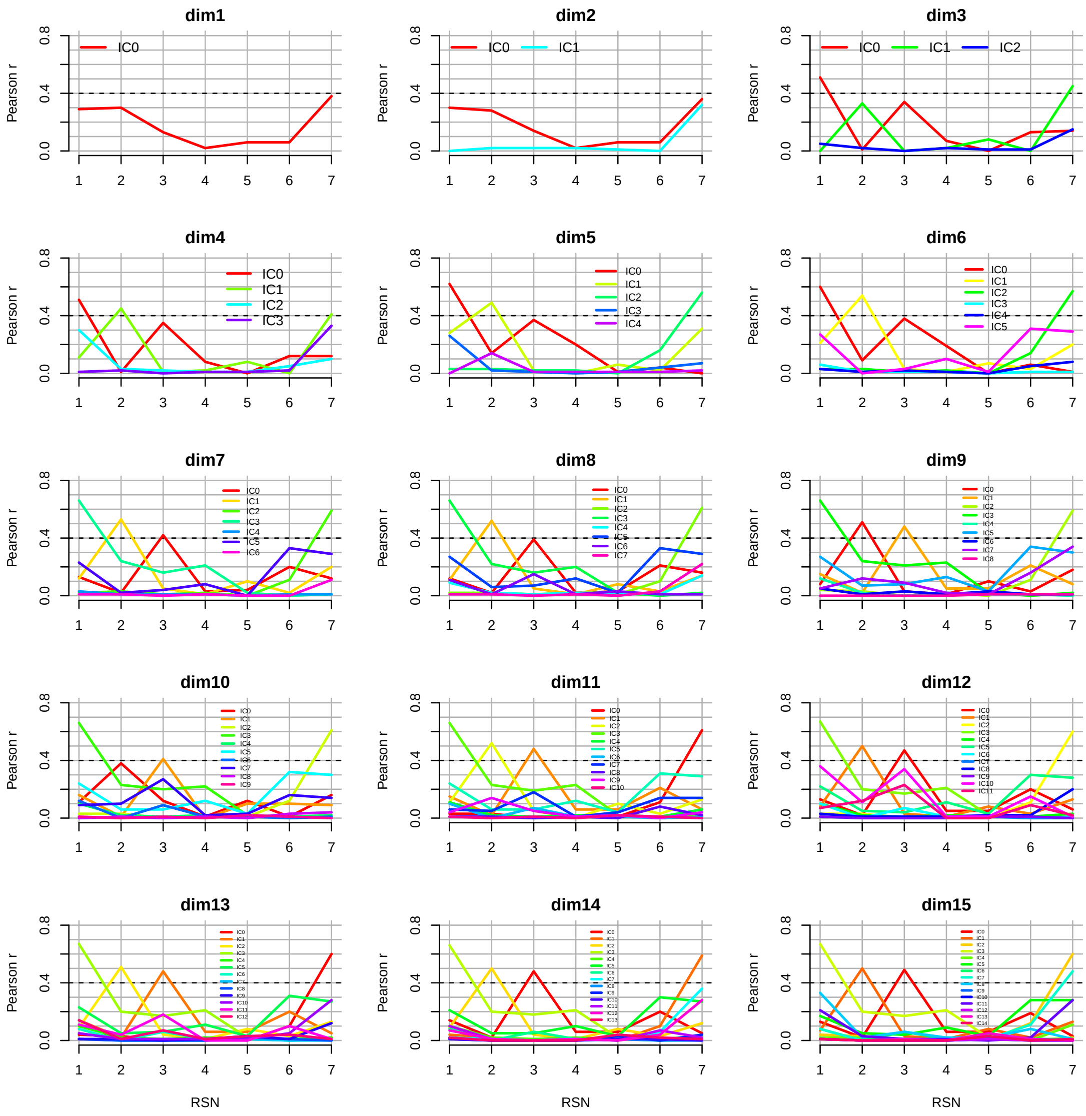


Figure 3: Correlations between independent components derived from srICA at dimensions 1 to 15 and the 7 different resting-state networks. Note how for most dimensions, ICs (and their corresponding whole-brain group level FC maps) show strong correlation (r > 0.4) with network 1 (visual), 2 (somatomotor), 3 (dorsal attention), or 7 (default mode), and that dimension 7 is the lowest dimension where a set of ICs shows high sensitivity and specificity with one and only one resting-state network. See main text for further details.

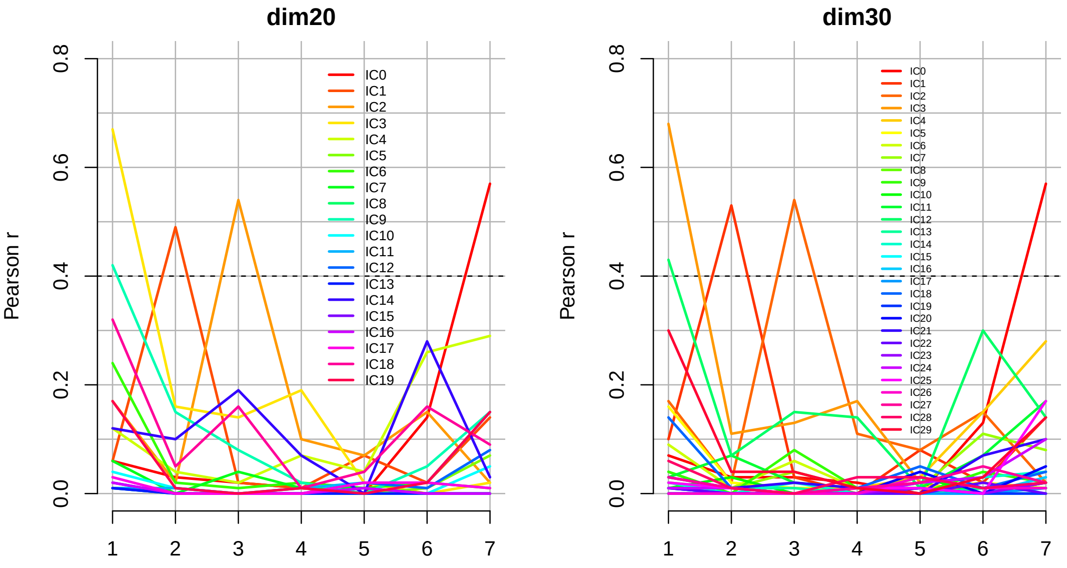


Figure 4: Correlations between independent components derived from srICA at dimensions 20 and 30 and the 7 different resting-state networks. Note how even at increased dimensions the four networks are detected with high reliability.

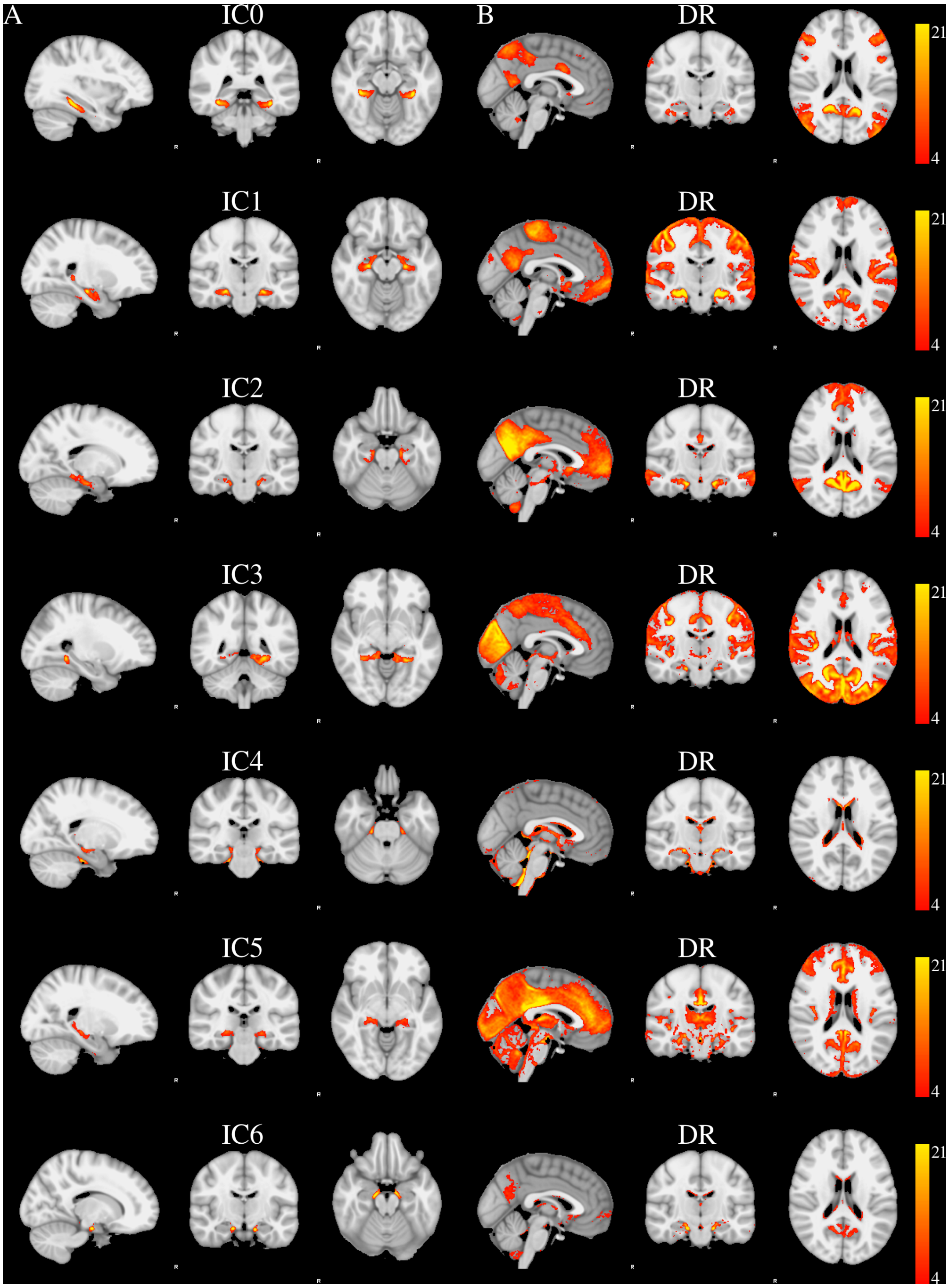


Figure 5: Overview of results from spatially restricted group ICA (left column) and Dual regression (right column) using dimension = 7 for all detected ICs in the test dataset. IC0 - IC3 were identified as having strong and unique correlations with resting-state networks. Note by looking at DR column how IC4 and IC5 appear to detect non-BOLD signals in regions around the brainstem and that IC6 does not show strong signal in any region.

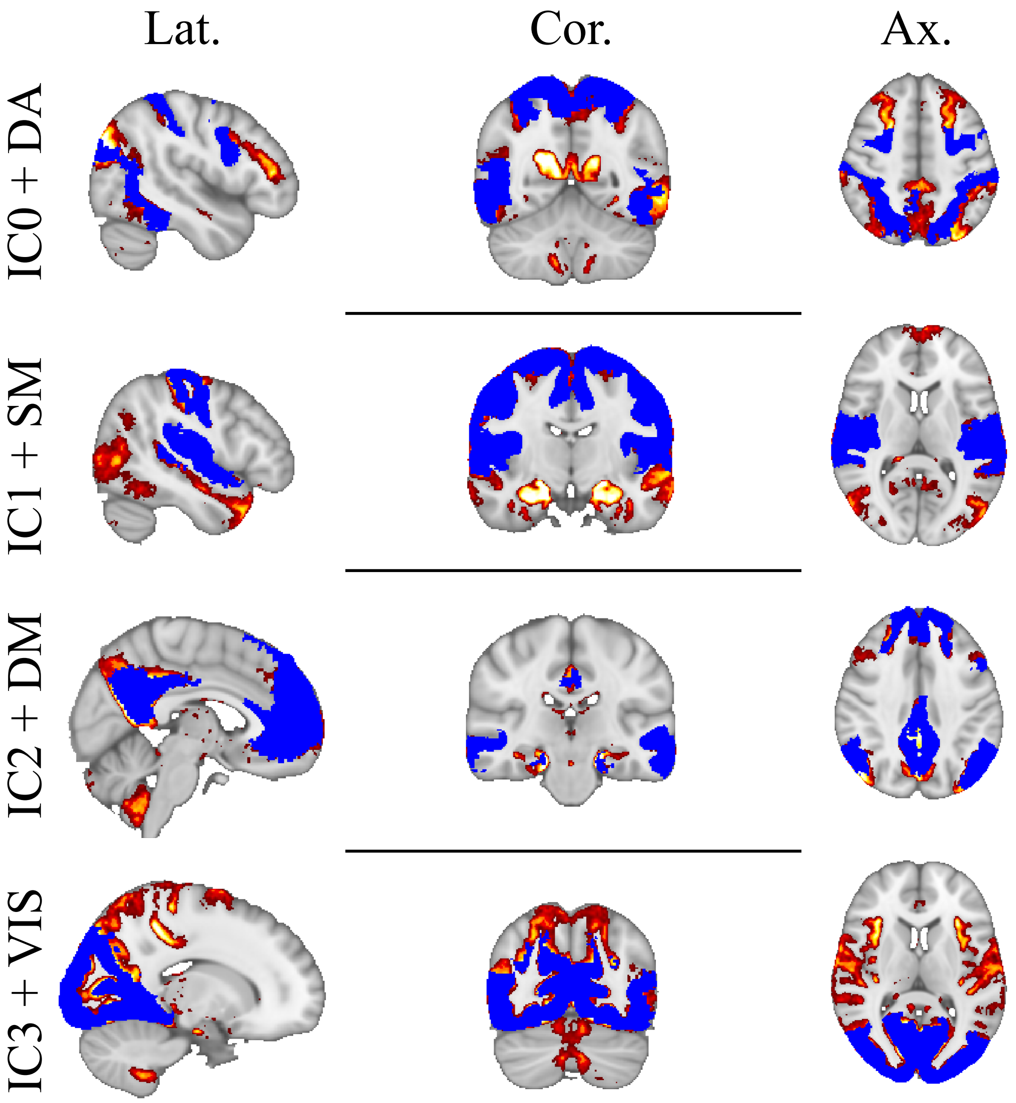


Figure 6: Overlap between the obtained whole brain FC maps corresponding to each MTL cluster (IC0 to IC3) and the corresponding reference network from Yeo (in blue). Note the high degree of overlap between the obtained network and the reference network and that the Yeo networks do not include subcortical regions. Lat = Lateral, Cor = Coronal, Ax = Axial, DA = Dosral Attention, SM = Somatomotor, DM = Default Mode, VIS = Visual, IC = Independent Component.

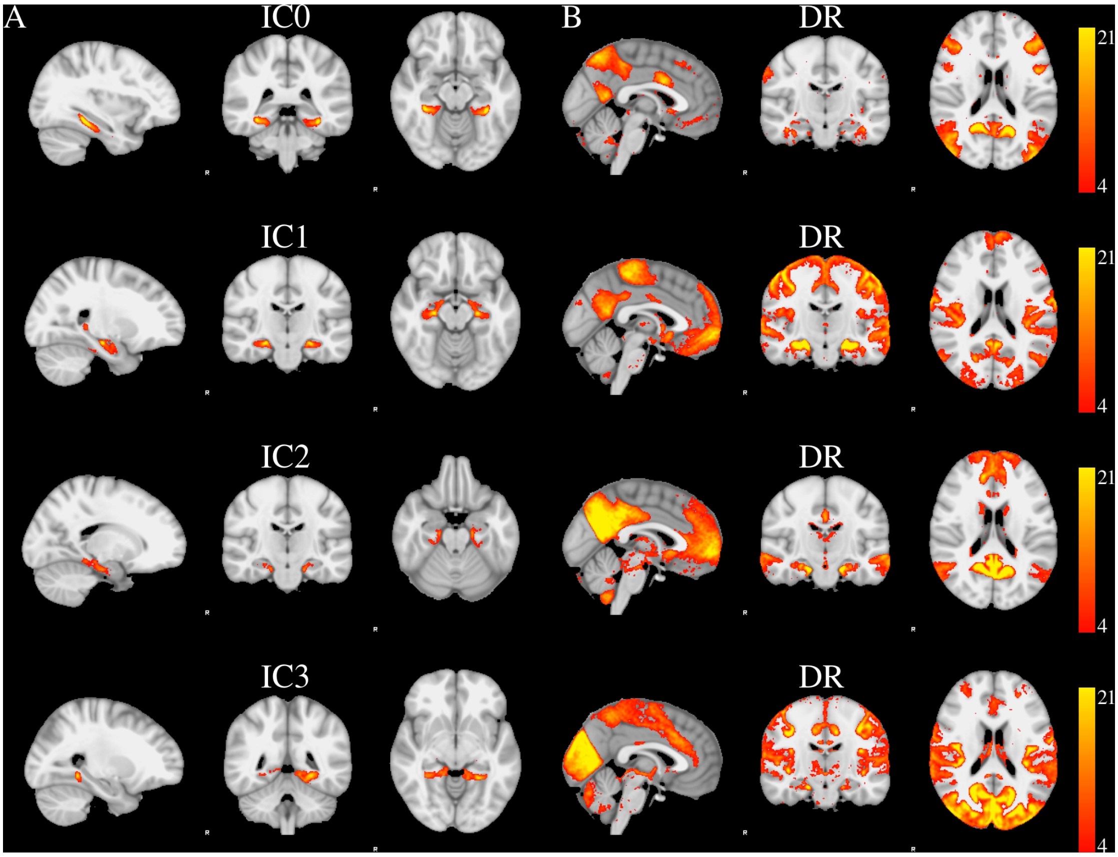


Figure 7: Overview of results from spatially restricted group ICA (left column) and Dual regression (right column) using dimension = 7 for the four ICs associated with resting-state networks in the validation dataset. Note the high similarity between the obtained functional connectivity in this validation dataset and the test dataset (cf., Figure S5).
